# Characterization of Antibiotic and Biocide Resistance Genes and Virulence Factors of *Staphylococcus* Species Associated with Bovine Mastitis in Rwanda

**DOI:** 10.3390/antibiotics9010001

**Published:** 2019-12-18

**Authors:** Fruzsina Irén Antók, Rosa Mayrhofer, Helene Marbach, Jean Claude Masengesho, Helga Keinprecht, Vedaste Nyirimbuga, Otto Fischer, Sarah Lepuschitz, Werner Ruppitsch, Monika Ehling-Schulz, Andrea T. Feßler, Stefan Schwarz, Stefan Monecke, Ralf Ehricht, Tom Grunert, Joachim Spergser, Igor Loncaric

**Affiliations:** 1 Institute of Microbiology, University of Veterinary Medicine, 1010 Vienna, Austria; fruzsi.antok@gmail.com (F.I.A.); rosa_m@gmx.at (R.M.); helene.marbach@vetmeduni.ac.at (H.M.); monika.ehling-schulz@vetmeduni.ac.at (M.E.-S.); tom.grunert@vetmeduni.ac.at (T.G.); joachim.spergser@vetmeduni.ac.at (J.S.); 2 New Vision Veterinary Hospital, Musanze, Rwanda; maceclau2@gmail.com (J.C.M.); helgakeinprecht@icloud.com (H.K.); nyirimbugavedaste@gmail.com (V.N.); owfischer@aol.com (O.F.); 3 Institute of Medical Microbiology and Hygiene, Austrian Agency for Health and Food Safety, 1010 Vienna, Austria; sarahlepuschitz@gmail.com (S.L.); werner.ruppitsch@ages.at (W.R.); 4 Institute of Microbiology and Epizootics, Centre for Infection Medicine, Department of Veterinary Medicine, Freie Universität Berlin, 10115 Berlin, Germany; andrea.fessler@fu-berlin.de (A.T.F.); stefan.schwarz@fu-berlin.de (S.S.); 5 Leibniz Institute of Photonic Technology (IPHT), 07743 Jena, Germany; stefan.monecke@leibniz-ipht.de (S.M.); ralf.ehricht@leibniz-ipht.de (R.E.); 6 InfectoGnostics Research Campus, 07743 Jena, Germany; 7 Institute for Medical Microbiology and Hygiene, Technical University of Dresden, 01307 Dresden, Germany; 8 Friedrich Schiller University Jena, Institute of Physical Chemistry, 07743 Jena, Germany

**Keywords:** *Staphylococcus* species, *Staphylococcus aureus*, bovine mastitis, antibiotic resistance, *spa* typing, FTIR spectroscopy, capsule serotyping, MLST, whole-genome sequencing, *dru* typing

## Abstract

The present study was conducted from July to August 2018 on milk samples taken at dairy farms in the Northern Province and Kigali District of Rwanda in order to identify *Staphylococcus* spp. associated with bovine intramammary infection. A total of 161 staphylococcal isolates originating from quarter milk samples of 112 crossbred dairy cattle were included in the study. Antimicrobial susceptibility testing was performed and isolates were examined for the presence of various resistance genes. *Staphylococcus aureus* isolates were also analyzed for the presence of virulence factors, genotyped by *spa* typing and further phenotypically subtyped for capsule expression using Fourier Transform Infrared (FTIR) spectroscopy. Selected *S. aureus* were characterized using DNA microarray technology, multi-locus sequence typing (MLST) and whole-genome sequencing. All *mecA*-positive staphylococci were further genotyped using *dru* typing. In total, 14 different staphylococcal species were detected, with *S. aureus* being most prevalent (26.7%), followed by *S. xylosus* (22.4%) and *S. haemolyticus* (14.9%). A high number of isolates was resistant to penicillin and tetracycline. Various antimicrobial and biocide resistance genes were detected. Among *S. aureus*, the Panton–Valentine leukocidin (PVL) genes, as well as bovine leukocidin (LukM/LukF-P83) genes, were detected in two and three isolates, respectively, of which two also carried the toxic shock syndrome toxin gene *tsst-1* bovine variant. t1236 was the predominant *spa* type. FTIR-based capsule serotyping revealed a high prevalence of non-encapsulated *S. aureus* isolates (89.5%). The majority of the selected *S. aureus* isolates belonged to clonal complex (CC) 97 which was determined using DNA microarray based assignment. Three new MLST sequence types were detected.

## 1. Introduction

Bovine mastitis is an important disease that affects the dairy sector and is one of the economically most important diseases worldwide [1]. In Rwanda, it has a significant relevance because livestock production is rapidly increasing [2]. One reason is that milk consumption and the demand for dairy products are increasing with the rapid growth of the human population, from 3 million to 12 million people [3] in the last 60 years.

Mastitis is an inflammation of the udder tissue and the mammary gland. It is usually caused by bacteria invading through the teat canal. There are two types of mastitis: clinical and subclinical. While cows with clinical mastitis show severe symptoms (e.g., fever, hot, painful and swollen udder) and have visible changes in their milk (e.g., change of colour and consistency), cows with subclinical mastitis produce less milk and have higher somatic cell counts in their milk [1]. The California Mastitis Test (CMT) is a useful onsite method to confirm a bovine intramammary infection [4].

Staphylococci are the leading cause of mastitis [5,6], with *S. aureus* considered to be a major pathogen associated with clinical mastitis and often-recurrent subclinical mastitis, even in well-managed dairy herds. The primary mode of transmission is from cow-to-cow [1]. Coagulase-negative *Staphylococcus* spp. (CoNS) are a heterogeneous group and are also known as common pathogens involved in bovine mastitis. CoNS are primarily derived from the environment and are usually associated with a moderate infection [1]. 

In Rwanda, udder infections are associated with contamination via hand-to-cow contact, clothing, and other materials because hand milking is common. Poor milking hygiene is a risk factor not only for mastitis, but also for teat-end damage [7]. Reduced milk production, high veterinary costs, as well as prolific bacterial and antimicrobial contamination are the consequences of mastitis which can result in significant economic losses for the farmers [8]. Recently, the Government of Rwanda launched a development program, called Rwanda Vision 2020, with the main goal of transforming the country into a knowledge-based middle-income country by modernizing its agriculture and livestock production [2]. Public veterinary services in Rwanda are provided by district and sector veterinary officers. They have a limited capacity to support dairy farmers. Often, veterinary service workers receive poor training in dairy management and are not equipped with adequate transportation to visit farms (approximately 3200 cattle/veterinary officers). Overall, access to veterinary services in rural areas is less developed compared to urban areas [9].

In 2015, the first private animal clinic was established in the district of Musanze, called the New Vision Veterinary Hospital (NVVH), to improve animal welfare and to provide veterinary services (clinical and laboratory) as well as education based on collaboration with local and foreign universities and organizations. 

Nevertheless, the farmers’ access in Rwanda to veterinary drugs including antibiotics is possible through local pharmacies [9]. A recent report explained that in parts of the country, high usage of antibiotics in farm animals has become a common practice [9]. In a cross-sectional survey, the use of antibiotics in farm animals was declared by the majority of respondents (97.4%), mainly for disease prevention and growth promotion. More than half of the farmers (55.6%) were reported to use non-prescribed antibiotics in animals. Although policies and laws regulating the antibiotic use in humans and animals exist in Rwanda, antibiotics can be purchased without any medical or veterinary prescription [9]. The irrational use of antibiotics in humans and animals is highly related to the increase of antibiotic-resistant bacteria worldwide, including many classes of antimicrobial agents used in the veterinary field [10].

A recent study conducted in a hospital in Kigali, Rwanda assessing the antimicrobial susceptibility patterns of bacteria from human patients, showed a high prevalence of antimicrobial resistance, also among *Staphylococcus* spp. [11]. However, there is very limited information on the antimicrobial susceptibility pattern of bacteria isolated from milk samples obtained from cases of bovine mastitis in Rwanda. Recently, two studies showed a high prevalence of mastitis in the Northern Province and the peri-urban areas of Kigali [12,13], but characterization of causative agents and antimicrobial susceptibility testing, both phenotypic and genotypic, have not been performed. Thus, the present study aims to fill these gaps by fully characterizing a collection of bovine staphylococci associated with clinical and subclinical mastitis from the Northern Province and Kigali the District of Rwanda. 

## 2. Results

From 303 CMT-positive milk samples collected from 112 crossbred milking cows, 161 non-repetitive staphylococcal isolates comprising 14 staphylococcal species were recovered: *S. aureus* (*n =* 43), *S. xylosus* (*n* = 36), *S. haemolyticus* (*n* = 24), *S. sciuri* (*n* = 14), *S. chromogenes* (*n* = 10), *S. saprophyticus* (*n* = 9), *S. epidermidis* (*n* = 8), *S. succinus* (*n* = 5), *S. capitis* (*n* = 3), *S. hominis* (*n* = 2), *S. devriesei* (*n* = 2), *S. auricularis* (*n* = 2), *S. equorum* (*n* = 2), and *S. simulans* (*n* = 1). 

### 2.1. Antimicrobial Susceptibility Testing

All 161 isolates were susceptible to rifampicin, linezolid, and gentamicin. All but two were susceptible to cefoxitin and chloramphenicol. A high number of the isolates was resistant to penicillin (*n* = 73, 45.3%) and tetracycline (*n* = 63, 39.1%) (Table 1 and Table 2). Twenty-three isolates were resistant to clindamycin, ten to erythromycin, and six isolates were resistant to trimethoprim-sulfamethoxazole (Table 1 and Table 2). 

The detection of resistance genes confirmed the phenotypic resistance profiles of the respective isolates, detecting *bla*Z (*n* = 73, 45.3%), *tet*(K) (*n* = 45, 71.4%), both *tet*(K) and *tet*(L) (*n* = 17, 27.0%) and all three *tet*(K), *tet*(L) and *tet*(O) (*n* = 1, 1.6%). Clindamycin-resistant isolates carried the following resistance genes: *erm*(C) (*n* = 8, 34.8%), *vga*(A) (*n* = 2, 8.7%), *erm*(44) (*n* = 2, 8.7%), *sal*(A) (*n* = 2, 8.7%), both *vga*(A) and *sal*(A) (*n* = 2, 8.7%), both *erm*(C) and *sal*(A) (*n* = 1, 4.3%), both *sal*(A) and *erm*(44) (*n* = 1, 4.3%) and all three *vga*(A), *sal*(A) and *lnu*(A) (*n* = 2, 8.7%). In the erythromycin-resistant isolates, two macrolide resistance genes were present: *erm*(C) (*n* = 6), and *msr*(A) (*n* = 4), whereas the trimethoprim-sulfamethoxazole-resistant isolates carried both *dfrA* (also known as *dfrS1*) and *dfrD* genes (*n* = 1), both *dfrD* and *dfrG* genes (*n* = 3) and all three *dfrA*, *dfrD* and *dfrG* genes (*n* = 2). Two isolates were resistant to chloramphenicol, which was associated with the presence of *fexA* in a *S. xylosus* and *cat*_pC221_ in a *S. saprophyticus* isolate. The streptomycin resistance gene *str* was detected in all 161 isolates, but its presence was not always associated with a higher MIC value (i.e., >8 mg/L) [14] (Table 1 and Table 2).

The *mecA* gene was detected in cefoxitin-resistant *S. hominis* and *S. sciuri*, whereas the *mecC* gene could not be identified. One *dru* type (dt10cz) was detected in a *S. hominis* isolate, but the other *mecA*-positive isolate was not *dru*-typeable.

None of the tested isolates carried the genes *erm*(A), *erm*(B), *erm*(F), *erm*(T), *erm*(43), *erm*(33), *Isa*(B), *vga*(A)_v_ *vga*(C), *vga*(E), *vga*(E)*v, dfrK, tet*(M), *ant(6’)-la, cfr, cat*_pC194_, or *cat*_pC223_.

### 2.2. Metal and Biocide Resistance Testing

Biocide resistance profiling revealed that 33 isolates carried the *smr* gene, most frequently the species *S. haemolyticus* (*n* = 7), *S. epidermidis* (*n* = 6), *S. xylosus* (*n* = 6) and *S. aureus* (*n* = 4). Seventeen isolates carried the *qacAB* gene, where the predominant species were *S. haemolyticus* (*n* = 4), *S. epidermidis* (*n* = 3), *S. aureus* (*n* = 3), *S. xylosus* (*n* = 2) and *S. hominis* (*n* = 2). Furthermore, the presence of the following metal resistance genes was confirmed: *cadD* (*n* = 25), *copB* (*n* = 27) and *arsA* (*n* = 21). The most prevalent species, which carried the *cadD* gene, was *S. haemolyticus* (*n* = 8), followed by *S. xylosus* (*n* = 5) and *S. epidermidis* (*n* = 4). A significant carriage rate of *copB* was shown by *S. saprophyticus* (*n* = 7) and *S. xylosus* (*n* = 7). The *arsA* gene was mostly detected in the species *S. haemolyticus* (*n* = 6), *S. epidermidis* (*n* = 4) and *S. saprophyticus* (*n* = 3). However, none of the isolates carried the *czrC* gene (Table 1 and Table 2) and all *S. aureus* isolates were negative for metal resistance genes.

### 2.3. Additional Characterization of S. aureus Isolates

Among *S. aureus*, the *lukS-PV* and *lukF-PV* genes coding for the Panton–Valentine leukocidin (PVL) were detected in two isolates, the bovine leukocidin gene *lukM/lukF-P83* was present in three isolates. The *tsst-1* gene was detected in two isolates and was solely found in combination with enterotoxin genes. The enterotoxin genes *sei* (*n* = 2), *sem* (*n* = 3), *sen* (*n* = 2), *seo* (*n* = 3) and *seu* (*n* = 2), that belonged to the *egc* cluster, and *sec* (*n* = 2) were detected. Staphylococcal enterotoxin genes *sea*, *seb*, *sed*, *see*, *seg*, *seh*, *sej*, *sek*, *sel*, *seq*, *ser* and the gene for the enterotoxin like protein CM14 could not be detected in the *S. aureus* isolates (Table 2). 

Ten different *spa* types were identified among the tested isolates. The *spa* type t1236 (*n* = 18) was predominant, followed by t10103 (*n* = 5), t380 (*n* = 4) and t9432 (*n* = 4), t458 (*n* = 4), t355 (*n* = 2) and singletons t2112 and t1398. Two new *spa* types were detected: t18835 (*n* = 2, repeat order 26-23-34-34-34-34-33-34) and t18853 (*n* = 1, repeat order 04-20-17-24-17).

**Figure 1:**
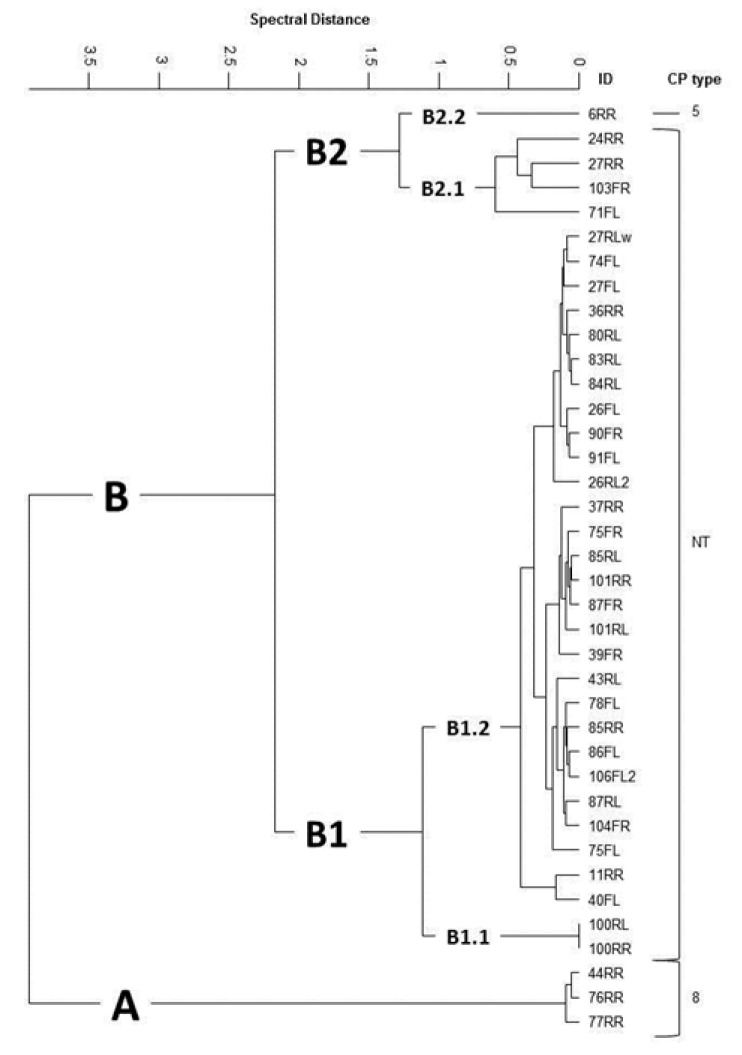
FTIR spectroscopy-based cluster of *S. aureus* isolated from quarter milk samples of cows with mastitis. CP = capsule type; NT = none typeable.

FTIR-based capsule serotyping revealed a high prevalence of non-encapsulated *S. aureus* isolates (*n* = 34; 89.5%) and the remaining isolates produced a capsule of either serotype 8 (CP8, *n* = 3) or 5 (CP5, *n* = 1). Hierarchical cluster analysis of spectral FTIR data grouped the *S. aureus* isolates into two main clusters (A; *n* = 3 and B; *n* = 35; Figure 1). Cluster A could be assigned to CP 8 while non-typeable (NT) isolates were grouped into the main cluster B, except one isolate assigned to CP5 (B2.2). All NT isolates were found to harbour either the *cap8*- (B2.1, *n* = 4) or *cap5*-specific allele (B1.1, *n* = 2 and B1.2, *n* = 28). No association between the origin of the samples and the FTIR cluster alignment was detectable.

Among the selected *S. aureus* isolates examined using DNA microarray and whole-genome sequencing, different resistance genes (*blaZ*, *erm*(C), *tet*(K)) and virulence genes (*hla*, *hlb*, *hld*, *lukD*, *lukE*, *lukM*, *lukF-P83*, *icaA*, *icaC*, *icaD*, *bap*, *clfA*, *clfB*, *fib*, *can*, *fnbA*, *fnbB*, *sasG*) could be found (Table 2). Four different clonal complexes (CC) were identified. Here, the CC97 isolates (*n* = 14) clustered into FTIR cluster B1, the CC3591 isolates (*n* = 4) into clusters A and B2.1, the CC3666 isolates (*n* = 2) into cluster B2.1 and one isolate of CC152 into cluster B2.2. Three *S. aureus* that were selected for MLST revealed the new sequence types ST5475 (199-805-44-430-447-192-733), ST5476 (199-806-741-2-447-192-734) and ST5477 (6-55-45-2-109-14-741).

## 3. Discussion

Clinical and subclinical mastitis can be one of the serious consequences of poor milking hygiene [5,7]. Previous studies have shown that the prevalence of mastitis within the East African region is high and that CoNS are common pathogens in bovine mastitis [5,15,16,17]. This finding was also confirmed in this study.

In the present study, *S. aureus* was the predominant *Staphylococcus* spp., which is in accordance with studies from other countries in that region, such as Tanzania, and Kenya [16,18]. Another study from Uganda showed that the predominant *Staphylococcus* spp. were from the CoNS group, but they were not further characterized to the species level [15]. Among CoNS, *S. chromogenes*, *S. haemolyticus*, *S. epidermidis*, *S. simulans* and *S. xylosus* are usually the most common isolated species associated with bovine mastitis [19,20]. However, distribution of CoNS species has shown to be herd-specific and influenced by different management practices that can vary between countries [1,20]. 

Penicillin resistance is probably the best known antimicrobial resistance property of *S. aureus* and its frequency in the current study is in accordance with other studies that examined antibiotic susceptibility patterns of staphylococci isolated from cases of bovine mastitis in other parts of Africa as well as in Germany and Finland [16,21,22,23,24]. Penicillin is a routinely used antimicrobial agent for the prevention and treatment of mastitis in dairy cows in Rwanda [9] and the *blaZ* gene was present in all 73 penicillin-resistant *Staphylococcus* spp. isolates (100%) in the current study. This gene encodes a narrow-spectrum β-lactamase which confers penicillin resistance [10,25].

Tetracycline belongs to the broad-spectrum antimicrobial agents and is also an often-used antimicrobial agent in farm animals in Rwanda [9]. Resistance to tetracyclines is frequently mediated by the genes *tet*(K) and *tet*(L), which code for active efflux mechanisms, and occasionally by *tet*(M) and *tet*(O), which encode ribosome-protective proteins [10]. In the present study, *tet*(K) was found in all tetracycline-resistant staphylococci (100%), followed by *tet*(L) (28.6%) and *tet*(O) (1.6%), while *tet*(M) was not detected in any of the tetracycline-resistant isolates. In a study from Tunisia, 10.3% of the staphylococcal isolates (*n* = 68) showed resistance to tetracycline and this resistance was always encoded by the *tet*(K) gene [26]. In another study from Germany, the *tet*(M), *tet*(K) and *tet*(L) genes were investigated among resistant *S. aureus* sisolates, originating from cases of bovine clinical mastitis (*n* = 25) and from farm personnel (*n* = 2), and *tet*(M) was found in 100%, *tet*(K) in 92.6% and *tet*(L) in 40.7% of the isolates [23].

Two *S. haemolyticus* and one *S. xylosus* isolate exhibited phenotypic resistance to clindamycin although a corresponding resistance gene was not detected. Whole genome sequencing of these isolates in a future study will hopefully clarify the genetic basis for the observed lincosamide resistance. Another problem detected in this study was the phenotypic assessment of streptomycin resistance. All isolates carried the resistance gene *str*, but MICs to streptomycin varied between ≤4 and 32 mg/L. Neither CLSI, nor EUCAST provide clinical breakpoints for streptomycin and staphylococci. The sequenced *str* amplicons obtained from staphylococcal isolates with low streptomycin MICs as well as from those with high streptomycin MICs did not differ in their sequences (author’s own observation). Again, whole genome approaches may help to clarify the situation.

Quaternary ammonium compounds (QACs)-based antiseptics are frequently used worldwide and this prevailing usage can lead to bacterial resistance against these substances [27,28]. In the current study, the antiseptic resistance genes *qacAB* and *smr* were examined. The *smr* gene was found more frequently than the *qacAB* genes. These results were similar to those of a study from Norway assessing the resistance to QACs in bacteria from milk samples obtained from 127 dairy cattle herds and 70 goat herds, where the *smr* gene was present in 64.2% and the *qacAB* gene in 28.5% of the isolates (*n* = 42) [28]. Studies about the bacterial resistance to QACs in staphylococci originating from bovine milk in Africa are scarce. One study from three African countries (Angola, São Tomé and Príncipe, Cape Verde), where a total of 301 *S. aureus* isolates were investigated, reported an intermediate prevalence for the *qacAB* gene (40.5%) and a low prevalence for the *smr* gene (3.7%) [29].

Many other substances with antimicrobial effects, including metal-containing compounds, are used in food-animal production, where they can contribute to the selection of isolates among staphylococcal species [30]. According to a study from 2017 on cattle production in East Rwanda, only 3.6% (*n* = 13) of the farmers practiced supplementary feeding [2]. However, in the present study, conducted in Northern parts of Rwanda and Kigali, 51 (31.5%) of the bacterial isolates carried at least one heavy metal resistance gene. Heavy metal resistance genes occurred most frequently in *S. haemolyticus* (*n* = 12) followed by *S. xylosus* (*n* = 11) and *S. saprophyticus* (*n* = 8). In another study, *S. haemolyticus* and *S. epidermidis* carried the most heavy metal resistance genes [31], but the isolates in the current study did not show a high rate of heavy metal resistance genes, which is possibly explained by the different geographical collection sites.

The vast majority of the collected *S. aureus* mastitis isolates in this study were non-encapsulated as shown by spectroscopic capsule serotyping. This is in concordance with several previous reports showing a high prevalence of non-encapsulated mastitis isolates in Argentina, USA and Austria [32,33,34]. Moreover, non-encapsulation was associated with high within-herd prevalence of *S. aureus*-based persistent, contagious bovine intramammary infections [35]. Indeed, this study provides further evidence that loss of capsule expression is a key phenotypic feature associated with bovine mastitis, a primarily chronic infection [36]. Out of the 38 FTIR-typed isolates, 22 were selected for clonal complex (CCs) identification using DNA microarray-based technology and three of them (two CC3591 and one CC3666) were genotyped by MLST. The four CCs (CC97, CC3591, CC3666, CC152) identified were relatively distinctive for one of the FTIR clusters, also seen by Kümmel et al. in 2016 [34], though no connection to one particular farm could be found. Most isolates were assigned to the common bovine lineage CC97, indicating predominance of this cattle-adapted clone, which has already been reported from bovine mastitis cases worldwide including Europe, Japan, Algeria, and South Africa [37,38,39,40].

The most predominant *spa* type among *S. aureus* in the present study was t1236. This is a *spa* type within ST97 and associated with CC97 along with the other *spa* types t2112, t380, and t10103, commonly found among *S. aureus* from neighbouring Uganda [41]. The *spa* type t1236 has also been detected among *S. aureus* from bovine milk in Japan, reported as ST97 [38]. The *spa* type t458, which was found in four isolates in the current study, has been detected in *S. aureus* from a case of bovine mastitis in China [42] and from bovine milk in Japan [38]. Many African studies (Democratic Republic of the Congo, Gabon, Ghana, Kenya, Nigeria and Uganda) reported the presence of *spa* type t355 in *S. aureus* from humans [43,44,45,46,47,48], which was also identified in three isolates in the current study.

Five *S. aureus* isolates carried PVL genes, which is of interest due to the common association with soft tissue and skin infections and the reported human to cow transmission of *S. aureus* [49,50]. The PVL genes code for proteins which are responsible for cytotoxic activity, especially leukocytes are affected [51]. The *lukS-PV* and *lukF-PV* genes (PVL genes) were mainly detected in *S. aureus* of human origin [52], but have also been reported in isolates from bovine mastitis cases in Africa suggesting human to cow transmission of the respective isolates [41,50]. These human-associated genes were also detected in two *S. aureus* ST152 isolates obtained from two cows kept in two different farms in this study (Table 2). The LukM/LukF-PV(P83) protein only kills bovine neutrophils and is common in *S. aureus* isolated from bovine mastitis [51,52]. In a study from North-Western Ethiopia, however, this bovine-related leukocidin was detected in a low percentage (4%) and the isolates did not belong to the common ST97 [50]. This was in line with the results of the current study where this gene was only present in three of the further selected *S. aureus* isolates, which belonged to ST5476 and to CC3591. Previous reports demonstrated that isolates belonging to ST97 may also be negative for the bovine-related leukocidin [38,53].

In the present study, the *tsst1* gene was detected in two isolates and further classified as bovine variant of *tsst1* which has been described in previous studies dealing with *S. aureus* associated with bovine mastitis [39,50,51,52,53,54,55].

## 4. Materials and Methods

### 4.1. Isolation and Identification of Staphylococci

Isolation of *Staphylococcus* spp. was conducted from July to August 2018 from CMT-positive milk samples originating from 112 crossbred dairy cows kept on 28 farms in the Northern Province and the Kigali District of Rwanda. Farms were selected for sampling based on farmers’ reports on decreased milk production of multiple cows. Before sampling, a short clinical check was performed on each selected cow, including palpation of the udder, examination of the milk and measuring the body temperature. Afterwards, CMT was performed, which can indicate the presence of mastitis [4]. Collected milk samples were transported to the microbiological laboratory of NVVH, and bacteriological analyses were performed. Milk samples were cultivated on blood agar (Blood Agar Base, Rapid Labs, UK) supplemented with 5% of defibrinated sheep blood. After incubation at 37 °C for 24 h, each colony representing a distinct colony morphotype, but showing typical staphylococcal colony appearance, was regrown on the same medium. Pure staphylococcal cultures were stored at 4 °C until they were transported to the diagnostic laboratory of the Institute of Microbiology at the University of Veterinary Medicine, Vienna for further examination. All isolates were regrown on BD Columbia III agar plates with 5% Sheep Blood (Becton Dickinson, Heidelberg, Germany), and identified by matrix-assisted laser desorption-ionization time-of-flight mass spectrometry (MALDI-TOF MS) (Bruker Daltonik, Bremen, Germany). If MALDI-TOF MS yielded ambiguous results, *rpoB* gene sequencing was performed [56].

### 4.2. Antimicrobial Susceptibility Testing and Detection of Resistance Genes and SCCmec-Associated Direct Repeat Unit (dru) Typing

Antimicrobial susceptibility testing was performed by agar disk diffusion according to CLSI standards (CLSI, 2018) for the following antimicrobial agents (μg/disk): tetracycline (30), ciprofloxacin (5), erythromycin (15), clindamycin (2), penicillin (10 IU), cefoxitin (30), chloramphenicol (30), gentamicin (10), rifampicin (5), linezolid (30), and trimethoprim-sulfamethoxazole (1.25 + 23.75). In addition, minimum inhibitory concentrations (MICs) of streptomycin were established by the agar dilution method on Mueller–Hinton agar in serial twofold dilutions (4, 8, 16, and 32 μg/mL) in accordance with the CLSI document M7-A9 (CLSI, 2012). 

Staphylococcal DNA was extracted as described previously [57]. PCR was used to detect the presence of the following antibiotic resistance genes: *blaZ* (confers resistance to penicillins except isoxazolyl-penicillins) [25]; *mecA*, *mecC* (confer resistance to all penicillins and cephalosporins approved for veterinary use) [58]; *erm*(A), *erm*(B), *erm*(C), *erm*(F), *erm*(T), *erm*(33), *erm*(43), and *erm*(44) (confer resistance to macrolides, lincosamides, and streptogramin B), *vga*(A), *vga*(A)_v_, *vga*(C), *vga*(E), *vga*(E)*v* and *sal*(A) (confer resistance to streptogramin A, lincosamides and pleuromutilins); *Isa*(B) and *Inu*(A) (confer elevated MICs or resistance to lincosamides) [23,59,60,61,62,63,64,65,66,67,68]; *msr*(A) (confers resistance to macrolides and streptogramin B) [57]; *cfr* (confers resistance to all phenicols, lincosamides, oxazolidinones, pleuromutilins, and streptogramin A) [69]; *fexA* (confers resistance to all phenicols) [69]; *cat*_pC194_, *cat*_pC221_, and *cat*_pC223_ (confer resistance to non-fluorinated phenicols, e.g. chloramphenicol) [70]; *ant*(6′)*-Ia* and *str* (confer resistance to the aminoglycoside streptomycin) [14]; *dfrA*, *dfrD*, *dfrG*, and *dfrK* (confer resistance to trimethoprim) [57,71]; *tet*(K) and *tet*(L) (confer resistance to tetracyclines except minocycline and glycylcyclines) [57]; *tet*(O) and *tet*(M) (confer resistance to tetracyclines, including minocycline, but excluding glycylcyclines) [72].

PCRs targeting *qacAB* (confers high-level resistance to antiseptics) and *smr* (confers low-level resistance to antiseptics) genes were performed as previously described [27]. Furthermore, PCRs were performed for detecting the presence of the following heavy metal resistance genes: *cadD*, *copB*, *arsA* and *czrC* [30,31].

The *mecA*-positive isolates were further examined by SCC*mec*-associated direct repeat unit (*dru*) typing [73].

### 4.3. Additional Characterization of S. aureus Isolates

All *S. aureus* isolates were examined by different PCRs targeting Panton–Valentine Leukocidin (PVL) genes, staphylococcal enterotoxins (SE), and the toxic shock syndrome toxin 1 (TSST1) as previously described [58]. Furthermore, *S. aureus* were genotyped by *spa* typing [57].

Using Fourier Transform Infrared (FTIR) spectroscopy, all isolates were further phenotypically subtyped based on their surface glyco structural composition that included the determination of the capsular polysaccharide (CP) expression [74,75]. On FTIR based clustering, 22 *S. aureus* isolates were selected and further analysed using DNA microarray-based technology to detect over 300 different target sequences including antimicrobial resistance and virulence-associated genes, species-specific genes, and SCC*mec*-associated genes [76]. Three isolates were genotyped using MLST as previously described [57]. In addition, whole-genome sequencing, as well as contig assembly and annotation, and comparative genomics were conducted as previously described using Seqsphere+ (Ridom, Münster, Germany) [77,78,79]. The same software was used for cgMLST [77]. The genomes of four *S. aureus* isolates were submitted under SUB6695668 in the NCBI BioProject database.

## 5. Conclusions

The present study is the first investigating not only the phenotypic but also the genotypic resistance to antimicrobial agents and biocides in *Staphylococcus* spp. isolated from cases of bovine mastitis in Rwanda. It improves our knowledge about the high diversity of *Staphylococcus* spp., their occurrence in the study area and about the presence of resistance genes. 

Due to the rising importance of the dairy production system in Rwanda, improvements in the prevention and treatment of bovine mastitis are critical to prevent misuse of antimicrobial agents and the increase of resistance to antimicrobial agents and biocides, which is in accordance with the ‘one world, one health’ principle [80].

## Figures and Tables

**Table 1:** Summarized molecular characterization, antimicrobial resistance and toxins profile of Coagulase-negative *Staphylococcus* isolates investigated.

Isolates	Species	Origin ^1^	Antimicrobial Resistance Profile			Biocide and Metal Resistance Genes
			Phenotype ^2^	MIC ^3^ of streptomycin	Genes Detected	
2FR	*S. chromogenes*	M 1		32 ^4^	*str*	
3RL	*S. haemolyticus*	M 1	ERY, CLI	32	*erm*(C), *str*	
4FR	*S. epidermidis*	M 1	PEN, TET	32	*blaZ, tet*(K), *tet*(L), *str*	*copB*, *qacAB*, *smr*
4RR1	*S. hominis*	M 1	BLA, FOX, ERY, TET, CIP	‹4	*blaZ*, *mecA*, *msr*(A), *tet*(K), *tet*(L), *str*	*cadD*, *arsA*, *qacAB*, *smr*
4RR2	*S. capitis*	M 1	PEN	‹4	*blaZ*, *str*	*cadD*, *arsA*, *qacAB*, *smr*
7FL	*S. chromogenes*	M 2	ERY, CLI	‹4	*erm*(C), *str*	
7RR	*S. epidermidis*	M 2	PEN, ERY, CLI, TET	32	*blaZ*, *erm*(C), *tet*(K), *tet*(L), *str*	*cadD*, *arsA*, *smr*
8RL	*S. haemolyticus*	M 2	ERY, CLI	32	*erm*(C), *str*	
13FLg	*S. xylosus*	M 3	PEN	‹4	*blaZ*, *str*	*cadD*, *copB*
13FLw	*S. xylosus*	M 3	PEN, TET	32	*blaZ*, *tet*(K), *tet*(L), *str*	*cadD*, *arsA*, *smr*
13FLw wh	*S. xylosus*	M 3	ERY	‹4	*msr*(A), *str*	
13RR	*S. xylosus*	M 3	ERY, CLI, CHL	‹4	*msr*(A), *fexA*, *str*	
14FL1	*S. equorum*	M 3		‹4	*str*	
17RR	*S. equorum*	M 4		‹4	*str*	*smr*
18RLw1	*S. epidermidis*	M 4	PEN, TET	32	*blaZ*, *tet*(K), *tet*(L), *str*	*cadD*, *arsA*, *qacAB*, *smr*
18RLw2	*S. haemolyticus*	M 4	PEN, TET	32	*blaZ*, *tet*(K), *tet*(L), *str*	*cadD*, *arsA*, *qacAB*, *smr*
18RLg	*S. haemolyticus*	M 4		‹4	*str*	*cadD*, *copB*, *arsA*, *smr*
18FL	*S. auricularis*	M 4		16	*str*	*copB*
24RLw	*S. xylosus*	M 5		‹4	*str*	*cadD*, *smr*
24RLg	*S. haemolyticus*	M 5		32	*str*	
25FLw	*S. hominis*	M 5	PEN	‹4	*blaZ*, *str*	*cadD*, *arsA*, *qacAB*, *smr*
25FLg	*S. xylosus*	M 5	PEN	‹4	*blaZ*, *str*	
25FL3	*S. xylosus*	M 5		‹4	*str*	*cadD*
25RR	*S. epidermidis*	M 5	PEN, TET	32	*blaZ*, *tet*(K), *str*	
25RRg	*S. sciuri*	M 5	CLI	‹4	*sal*(A), *str*	
26RL1	*S. xylosus*	M 6		‹4	*str*	*cadD*
26RRw	*S. xylosus*	M 6		‹4	*str*	
26RRg	*S. xylosus*	M 6		‹4	*str*	
27RLg	*S. xylosus*	M 6		‹4	*str*	
28FRg	*S. xylosus*	M 7		‹4	*str*	
30FL	*S. devriesei*	M 8	TET	16	*tet*(K), *str*	*arsA*
30RL	*S. devriesei*	M 8	PEN, TET	32	*blaZ*, *tet*(K), *str*	*arsA*
30FR	*S. chromogenes*	M 8	PEN, TET	‹4	*blaZ*, *tet*(K), *str*	
32FR	*S. chromogenes*	M 8		32	*str*	
33RL	*S. chromogenes*	M 8	PEN, TET	32	*blaZ*, *tet*(K), *str*	
33FR	*S. haemolyticus*	M 8		32	*str*	
34RLw	*S. haemolyticus*	M 9		32	*str*	*cadD*
35FR	*S. haemolyticus*	M 9		16	*str*	*arsA*
35RRg	*S. haemolyticus*	M 9		16	*str*	*arsA*
36FL	*S. haemolyticus*	M 9	TET	32	*tet*(K), *tet*(L), *str*	
38FL	*S. auricularis*	M 9		‹4	*str*	*cadD*
42FR	*S. haemolyticus*	M 11	TET	32	*tet*(K), *tet*(L), *str*	
43FRw	*S. xylosus*	M 11	TET	‹4	*tet*(K), *str*	*copB*
44FL	*S. xylosus*	M 11		‹4	*str*	
46FR	*S. epidermidis*	M 11	PEN	32	*blaZ*, *str*	*cadD*
47RRg	*S. chromogenes*	M 12		32	*str*	*qacAB*, *smr*
50RL	*S. sciuri*	M 12	CLI	‹4	*erm*(44), *str*	
50RR	*S. sciuri*	M 12	CLI	‹4	*erm*(44), *sal*(A) *str*	
51RR	*S. xylosus*	M 12	TET	‹4	*tet*(K), *str*	
52FL	*S. haemolyticus*	K	PEN, CLI, TET	32	*blaZ*, *erm*(C), *tet*(K), *tet*(L), *str*	*cadD*, *copB*, *qacAB*, *smr*
52FR	*S. haemolyticus*	K		‹4	*str*	*cadD*, *copB*, *arsA*
53FL	*S. haemolyticus*	K	PEN, CLI, TET	32	*blaZ*, *tet*(K), *str*	*copB*
53RL	*S. haemolyticus*	K	CLI, TET	32	*vga*(A), *sal*(A), *Inu*(A), *tet*(K), *tet*(L), *str*	*qacAB*, *smr*
53RR	*S. haemolyticus*	K	CLI, TET	32	*vga*(A), *sal*(A), *Inu*(A), *tet*(K), *tet*(L), *str*	*qacAB*, *smr*
54FR	*S. haemolyticus*	K	CLI	32	*vga*(A), *str*	
54RRw	*S. haemolyticus*	K	PEN, CLI, SXT, TET	32	*blaZ*, *dfrA*, *dfrD*, *tet*(K), *str*	
54RRg	*S. xylosus*	K		32	*str*	*smr*
55RR1	*S. epidermidis*	K	PEN, TET	‹4	*blaZ*, *tet*(K), *str*	*copB*, *arsA*, *qacAB*, *smr*
55RR2	*S. capitis*	K	PEN	‹4	*blaZ*, *str*	*copB*, *arsA*, *smr*
56RL	*S. sciuri*	K	CLI	‹4	*vga*(A), *sal*(A), *str*	
57FLw	*S. capitis*	K	PEN, TET	‹4	*blaZ*, *tet*(K), *tet*(L), *str*	*cadD*, *smr*
57FRw	*S. haemolyticus*	K	CLI, TET	32	*tet*(K), *tet*(L), *str*	*copB*, *smr*
58FL	*S. haemolyticus*	K	CLI, TET	32	*erm*(C), *sal*(A), *tet*(K), *tet*(L), *str*	*smr*
58FR	*S. haemolyticus*	K	CLI, TET	32	*vga*(A), *tet*(K), *tet*(L), *str*	
58RR	*S. xylosus*	K		‹4	*str*	
61RR	*S. xylosus*	K	SXT, TET	‹4	*dfrA*, *dfrD*, *dfrG*, *tet*(K), *tet*(L), *str*	*smr*
61RL	*S. xylosus*	K	TET	32	*tet*(K), *str*	*copB*, *smr*
62FR	*S. xylosus*	K		‹4	*str*	*copB*
62RR	*S. haemolyticus*	K		‹4	*str*	*cadD*
63RL	*S. sciuri*	K	PEN	‹4	*blaZ*, *str*	
64RR	*S. epidermidis*	K	PEN, SXT, TET	32	*blaZ*, *dfrA*, *dfrD*, *dfrG*, *tet*(K), *tet*(L), *tet*(O), *str*	*copB*, *arsA*, *smr*
65RL	*S. haemolyticus*	K	PEN, ERY, SXT, TET	32	*blaZ*, *msr*(A), *dfrD*, *dfrG*, *tet*(K), *str*	*cadD*, *copB*, *arsA*
66RL	*S. xylosus*	K	PEN, TET	‹4	*blaZ*, *tet*(K), *str*	*qacAB*
66RR	*S. epidermidis*	K	PEN, TET, TEC	32	*blaZ*, *tet*(K), *str*	*cadD*, *smr*
67RL	*S. chromogenes*	K		32	*str*	
68RL	*S. chromogenes*	K		32	*str*	
68RR	*S. xylosus*	K	PEN	‹4	*blaZ*, *str*	
70RLw	*S. simulans*	K	PEN	32	*blaZ*, *str*	*copB*
70FR	*S. sciuri*	K	FOX	‹4	*mecA*, *str*	
1stCowFL	*S. chromogenes*	M 13		‹4	*str*	
2ndCowRL	*S. xylosus*	M 13	TET	‹4	*tet*(K), *str*	
73RL	*S. sciuri*	M 14	PEN	‹4	*blaZ*, *str*	
73RR	*S. xylosus*	M 14		‹4	*str*	
78FR	*S. xylosus*	M 17		‹4	*str*	
78RL	*S. sciuri*	M 17	CLI	‹4	*vga*(A), *sal*(A), *str*	
81 RR	*S. haemolyticus*	M 18	PEN	‹4	*blaZ*, *str*	*cadD*
82RL	*S. sciuri*	M 18	CLI	‹4	*erm*(44), *str*	
82RR	*S. saprophyticus*	M 18	TET, CHL	4	*tet*(K), *cat*_pC221_, *str*	
84RR	*S. saprophyticus*	M 18	TET	‹4	*tet*(K), *str*	*copB*
85FR	*S. xylosus*	M 19	TET	8	*tet*(K), *str*	
85FL	*S. saprophyticus*	M 19	TET	8	tet(K), *str*	*copB*, *arsA*, *qacAB*
86FR	*S. saprophyticus*	M 19		‹4	*str*	*copB*
87FL	*S. saprophyticus*	M 19	TET	4	*tet*(K), *str*	*copB*
89FR	*S. sciuri*	M 20		‹4	*str*	
89RR	*S. xylosus*	M 20	PEN	‹4	*blaZ*, *str*	
94RR	*S. succinus*	M 21	PEN	‹4	*blaZ*, *str*	*copB*
94RL	*S. sciuri*	M 21	PEN	‹4	*blaZ*, *str*	
95FR	*S. xylosus*	M 21		‹4	*str*	
95RR	*S. xylosus*	M 21		‹4	*str*	
96FR	*S. xylosus*	M 21	TET	‹4	*tet*(K), *str*	*qacAB*
96RR	*S. xylosus*	M 21		‹4	*str*	
97RL	*S. sciuri*	M 21		‹4	*str*	
97RR	*S. xylosus*	M 21	SXT	‹4	*dfrD*, *dfrG*, *str*	
98RR	*S. succinus*	M 21	PEN	‹4	*blaZ*, *str*	*cadD*
99FR	*S. xylosus*	M 22		‹4	*str*	
99RL	*S. xylosus*	M 22		‹4	*str*	*copB*
103RR	*S. chromogenes*	M 22	PEN	32	*blaZ*, *str*	
104RR	*S. succinus*	M 23		‹4	*str*	*smr*
104RL	*S. succinus*	M 23	PEN	‹4	*blaZ*, *str*	*cadD*, *arsA*, *smr*
105RL	*S. succinus*	M 24		‹4	*str*	*cadD*, *smr*
106FL1	*S. saprophyticus*	M 24		‹4	*str*	*copB*
107RL	*S. saprophyticus*	M 25	PEN	16	*blaZ*, *str*	*copB*
108FL	*S. saprophyticus*	M 25	SXT	‹4	*dfrD*, *dfrG*, *str*	*arsA*
110RL	*S. xylosus*	M 26		‹4	*str*	*copB*, *smr*
110RR1	*S. saprophyticus*	M 26		‹4	*str*	*copB*, *arsA*
110RR2	*S. xylosus*	M 26		‹4	*str*	*copB*
111RL	*S. sciuri*	M 26	PEN, CLI	‹4	*sal*(A), *blaZ*, *str*	
113RL	*S. sciuri*	M 26		16	*str*	

^1^ Origin: M = Musanze Farm, K = Kigali Farm.^2^ Phenotype: PEN = penicillin; CIP = ciprofloxacin; CHL = chloramphenicol; CLI = clindamycin; ERY = erythromycin; SXT = trimethoprim-sulfamethoxazole; TET = tetracycline; FOX = cefoxitin, TEC = teicoplanin. ^3^ mg/L. ^4^ 32 or higher (mg/L).

**Table 2:** Summarized molecular characterization, antimicrobial resistance and toxin profile of the *Staphylococcus aureus* isolates investigated.

Isolates	Origin ^1^	CC ^2^	ST ^3^	*spa*	Antimicrobial Resistance Profile			Biocide and Metal Resistance Genes	Capsule Serotype ^7^	*cap* gene (*cap* 8)	*cap* gene (*cap* 5)	Hemolysins	Leukocidin (luk) Components	Biofilm-Associated Genes	Adhesion Factors	Superantigens
					Phenotype ^4^	MIC ^5^ of Streptomycin	Genes Detected									
1FR *	M 1		ST97	t1236	PEN	32 ^6^	*blaZ*, *str*		not tested	NEG ^8^	POS ^8^	*hla*, *hlb*, *hld*	NEG	*icaC*, *icaD*	*clfA*, *fib*, *fnbA*, *fnbB*, *sasG*	
6RR *	M 2	CC152	ST152	t458	ERY, CLI	32	*erm*(C), *str*		CP5	NEG	POS	*hla*, *hlb*, *hld*	*lukS-PV/lukF-PV*	*icaA*, *icaD*	*clfA*, *clfB*, *cna*, *fnbA*, *fnbB*	
11RR *	M 3		ST97	t1236	PEN	‹4	*blaZ*, *str*	*smr*	nt	NEG	POS	*hla*, *hlb*, *hld*	NEG	*icaC*, *icaD*	*clfA*, *fib*, *fnbA*, *fnbB*, *sasG*	
24RR *	M 5	CC3666	ST5477	t1236	PEN, TET	32	*blaZ*, *tet*(K), *tet*(L), *str*		nt	POS	NEG	*hla*, *hld*	*lukD*	*icaA*, *icaC*, *icaD*	*clfA*, *clfB*, *fib*, *fnbA*, *fnbB*, *sasG*	*tsst-1*, *sei*, *sem*, *sen*, *seo*, *seu*
26FR	M 6			t1236	PEN	32	*blaZ*, *str*		not tested	not tested	not tested	not tested	not tested	not tested	not tested	
26FL	M 6	CC97		t1236	PEN	16	*blaZ*, *str*		nt	NEG	POS	*hla*, *hlb*, *hld*	*lukD*, *lukE*	*icaA*, *icaC*, *icaD*	*clfA*, *clfB*, *fib*, *fnbA*, *fnbB*, *sasG*	
26RL2	M 6			nt^7^	PEN	32	*blaZ*, *str*		nt	not tested	not tested	not tested	not tested	not tested	not tested	
27FL	M 6			t1236	PEN	32	*blaZ*, *str*		nt	not tested	not tested	not tested	not tested	not tested	not tested	*sec*
27RLw	M 6	CC97		t1236	PEN	‹4	*blaZ*, *str*		nt	NEG	POS	*hla*, *hlb*, *hld*	*lukD*, *lukE*	*icaA*, *icaC*, *icaD*	*clfA*, *clfB*, *fib*, *fnbA*, *fnbB*, *sasG*	
27RR	M 6			t1398	TET	4	*tet*(K), *str*		nt	not tested	not tested	not tested	not tested	not tested	not tested	
36RR	M 9	CC97		t1236	PEN	32	*blaZ*, *str*		nt	NEG	POS	*hla*, *hlb*, *hld*	*lukD*, *lukE*	*icaA*, *icaC*, *icaD*	*clfA*, *clfB*, *fib*, *fnbA*, *fnbB*, *sasG*	
37RR	M 9			t9432	PEN, TET	‹4	*blaZ*, *tet*(K), *str*		nt	not tested	not tested	not tested	not tested	not tested	not tested	
39FR	M 10	CC97		t2112	PEN	8	*blaZ*, *str*		nt	NEG	POS	*hla*, *hlb*, *hld*	*lukD*, *lukE*	*icaA*, *icaC*, *icaD*	*clfA*, *clfB*, *fib*, *fnbA*, *fnbB*, *sasG*	
40FL	M 10	CC97		t1236	PEN, TET	32	*blaZ*, *tet*(K), *str*		nt	NEG	POS	*hla*, *hlb*, *hld*	*lukD*, *lukE*	*icaA*, *icaC*, *icaD*	*clfA*, *clfB*, *fib*, *fnbA*, *fnbB*, *sasG*	
43RL	M 11	CC97		t18835	PEN, TET	32	*blaZ*, *tet*(K), *str*		nt	NEG	POS	*hla*, *hlb*, *hld*	*lukD*, *lukE*	*icaA*, *icaC*, *icaD*	*clfA*, *clfB*, *fib*, *fnbA*, *fnbB*, *sasG*	
44RR	M 11	CC3591		t458		‹4	*str*	*smr*	CP8	POS	NEG	*hla*, *hlb*, *hld*	*lukM/lukF-PV (P83)*	*icaA*, *icaC*, *icaD*	*clfA*, *clfB*, *fib*, *cna*, *fnbA*	
63FL	K	CC152	ST152	t355	ERY, CLI	32	*erm*(C), *str*		not tested	NEG	POS	*hla*, *hlb*, *hld*	*lukS-PV/lukF-PV*	*icaA*, *icaD*	*clfA*, *clfB*, *cna*, *fnbA*, *fnbB*	
71FL	M 14	CC3591	ST5475	t355	TET	32	*tet*(K), *str*		nt	POS	NEG	*hla*, *hlb*, *hld*	NEG	*icaA*, *icaC*, *icaD*	*clfA*, *clfB*, *fib*, *cna*, *fnbA*	*sem*, *seo*
74FL	M 14			t1236	PEN, TET	32	*blaZ*, *tet*(K), *str*		nt	not tested	not tested	not tested	not tested	not tested	not tested	
75FR	M 15	CC97		t10103	PEN, TET	32	*blaZ*, *tet*(K), *str*		nt	NEG	POS	*hla*, *hlb*, *hld*	*lukD*, *lukE*	*icaA*, *icaC*, *icaD*	*clfA*, *clfB*, *fib*, *fnbA*, *fnbB*, *sasG*	
75FL	M 15			t1236	PEN, TET	32	*blaZ*, *tet*(K), *str*		nt	not tested	not tested	not tested	not tested	not tested	not tested	
76RR	M 16	CC3591	ST5476	t458		‹4	*str*		CP8	POS	NEG	*hla*, *hlb*, *hld*	*lukM/lukF-PV (P83)*	*icaA*, *icaC*, *icaD*	*clfA*, *clfB*, *fib*, *cna*, *fnbA*	
77RR	M 17	CC3591		t458		16	*str*		CP8	POS	NEG	*hla*, *hlb*, *hld*	*lukM/lukF-PV (P83)*	*icaA*, *icaC*, *icaD*	*clfA*, *clfB*, *fib*, *cna*, *fnbA*	
78FL	M 17	CC97		t1236	PEN, TET	32	*blaZ*, *tet*(K), *str*		nt	NEG	POS	*hla*, *hlb*, *hld*	*lukD*, *lukE*	*icaA*, *icaC*, *icaD*	*clfA*, *clfB*, *fib*, *fnbA*, *fnbB*, *sasG*	
80RL	M 18			t380	PEN, TET	32	*blaZ*, *tet*(K), *str*		nt	not tested	not tested	not tested	not tested	not tested	not tested	*sec*
82FL	M 18			t380	PEN, TET	‹4	*blaZ*, *tet*(K), *str*		not tested	not tested	not tested	not tested	not tested	not tested	not tested	
83RL	M 18	CC97		t380	PEN	32	*blaZ*, *str*		nt	NEG	POS	*hla*, *hlb*, *hld*	*lukD*, *lukE*	*icaA*, *icaC*, *icaD*	*clfA*, *clfB*, *fib*, *fnbA*, *fnbB*, *sasG*	
84RL	M 18			t380	PEN, TET	32	*blaZ*, *tet*(K), *str*		nt	not tested	not tested	not tested	not tested	not tested	not tested	
85RR	M 19	CC97		t1236	PEN, TET	32	*blaZ*, *tet*(K), *str*		nt	NEG	POS	*hla*, *hlb*, *hld*	*lukD*, *lukE*	*icaA*, *icaC*, *icaD*	*clfA*, *clfB*, *fib*, *fnbA*, *fnbB*, *sasG*	
85RL	M 19			t10103	PEN, TET	32	*blaZ*, *tet*(K), *str*	*qacAB*	nt	not tested	not tested	not tested	not tested	not tested	not tested	
86FL	M 19			t1236	PEN, TET	32	*blaZ*, *tet*(K), *str*		nt	not tested	not tested	not tested	not tested	not tested	not tested	
87FR	M 19			t10103	PEN, TET	32	*blaZ*, *tet*(K), *str*		nt	not tested	not tested	not tested	not tested	not tested	not tested	
87RL	M 19			t1236	PEN, TET	32	*blaZ*, *tet*(K), *str*		nt	not tested	not tested	not tested	not tested	not tested	not tested	
90FR	M 20			t9432	PEN, TET	32	*blaZ*, *tet*(K), *str*		nt	not tested	not tested	not tested	not tested	not tested	not tested	
90FL	M 20			t9432	PEN, TET	32	*blaZ*, *tet*(K), *str*		not tested	not tested	not tested	not tested	not tested	not tested	not tested	
91FL	M 20	CC97		t9432	PEN	32	*blaZ*, *str*		nt	NEG	POS	*hla*, *hlb*, *hld*	*lukD*, *lukE*	*icaA*, *icaC*, *icaD*	*clfA*, *clfB*, *fib*, *fnbA*, *fnbB*, *sasG*	
100RR	M 22	CC97		t1236	PEN, TET	‹4	*blaZ*, *tet*(K), *str*	*qacAB*	nt	NEG	POS	*hla*, *hlb*, *hld*	*lukD*, *lukE*	*icaA*, *icaC*, *icaD*	*clfA*, *clfB*, *fib*, *fnbA*, *fnbB*, *sasG*	
100RL	M 22	CC97		t1236	PEN, TET	‹4	*blaZ*, *tet*(K), *str*		nt	NEG	POS	*hla*, *hlb*, *hld*	*lukD*, *lukE*	*icaA*, *icaC*, *icaD*	*clfA*, *clfB*, *fib*, *fnbA*, *fnbB*, *sasG*	
101RR	M 22	CC97		t10103	PEN	32	*blaZ*, *str*		nt	NEG	POS	*hla*, *hlb*, *hld*	*lukD*, *lukE*	*icaA*, *icaC*, *icaD*	*clfA*, *clfB*, *fib*, *fnbA*, *fnbB*, *sasG*	
101RL	M 22			t10103	PEN	32	*blaZ*, *str*		nt	not tested	not tested	not tested	not tested	not tested	not tested	
103FR	M 22	CC3666		t18853	PEN, TET	32	*blaZ*, *tet*(K), *str*	*smr*	nt	POS	NEG	*hla*, *hlb*, *hld*	*lukD*	*icaA*, *icaC*, *icaD*	*clfA*, *clfB*, *fib*, *fnbA*, *fnbB*, *sasG*	*tsst-1*, *sei*, *sem*, *sen*, *seo*, *seu*
104FR	M 23			t1236	PEN, TET	32	*blaZ*, *tet*(K), *str*	*smr*	nt	not tested	not tested	not tested	not tested	not tested	not tested	
106FL2	M 24			t18835	PEN, TET	32	*blaZ*, *tet*(K), *str*	*qacAB*	nt	not tested	not tested	not tested	not tested	not tested	not tested	

^1^ Origin: M = Musanze Farm, K = Kigali Farm. ^2^ clonal complex. ^3^ sequence type. ^4^ Phenotype: PEN = penicillin; CIP = ciprofloxacin; CHL = chloramphenicol; CLI = clindamycin; ERY = erythromycin; SXT = trimethoprim-sulfamethoxazole; TET = tetracycline; FOX = cefoxitin, TEC = teicoplanin. ^5^ mg/L. ^6^ 32 or higher (mg/L); ^7^ Capsule serotype: nt = non-typable; CP5 = Serotype 5; CP8 = Serotype 8. ^8^ NEG = negative, POS = positive. * analysed by whole-genome sequencing.
